# Ventilation Weaning and Extubation Readiness in Children in Pediatric Intensive Care Unit: A Review

**DOI:** 10.3389/fped.2022.867739

**Published:** 2022-04-01

**Authors:** Elisa Poletto, Francesca Cavagnero, Marco Pettenazzo, Davide Visentin, Laura Zanatta, Fabrizio Zoppelletto, Andrea Pettenazzo, Marco Daverio, Claudia Maria Bonardi

**Affiliations:** ^1^Department of Woman's and Child's Health, University Hospital of Padua, Padua, Italy; ^2^Pediatric Intensive Care Unit, Department of Woman's and Child's Health, University Hospital of Padua, Padua, Italy

**Keywords:** weaning, mechanical ventilation, extubation, pediatric, children, pediatric intensive care unit

## Abstract

Ventilation is one of the most common procedures in critically ill children admitted to the pediatric intensive care units (PICUs) and is associated with potential severe side effects. The longer the mechanical ventilation, the higher the risk of infections, mortality, morbidity and length of stay. Protocol-based approaches to ventilation weaning could have potential benefit in assisting the physicians in the weaning process but, in pediatrics, clear significant outcome difference related to their use has yet to be shown. Extubation failure occurs in up to 20% of patients in PICU with evidences demonstrating its occurrence related to a worse patient outcome including higher mortality. Various clinical approaches have been described to decide the best timing for extubation which can usually be achieved by performing a spontaneous breathing trial before the extubation. No clear evidence is available over which technique best predicts extubation failure. Within this review we summarize the current strategies of ventilation weaning and extubation readiness evaluation employed in the pediatric setting in order to provide an updated view on the topic to guide intensive care physicians in daily clinical practice. We performed a thorough literature search of main online scientific databases to identify principal studies evaluating different strategies of ventilation weaning and extubation readiness including pediatric patients receiving mechanical ventilation. Various strategies are available in the literature both for ventilation weaning and extubation readiness assessment with unclear clear data supporting the superiority of any approach over the others.

## Introduction

Mechanical ventilation (MV) is a common procedure for patients admitted to the pediatric intensive care units (PICUs). Although MV is necessary and life-saving, it can be associated with complications such as ventilator associated pneumonia, cardiovascular disfunction, airway injury and patient's immobility (1, 2). The longer the MV, the higher the risk of morbidity, length of stay and mortality (3–5). To reduce the risks associated with a prolonged MV, the clinicians should aim to constantly optimize the ventilation weaning (VWe) process, thus increasing the

likelihood of a successful extubation. VWe is defined as “the gradual reduction of mechanical ventilatory support and the transfer of the respiratory control and the work of breathing back to the patient” (6). Traditionally, VWe was achieved by clinical judgement on a personal decision. Only in the last few years, protocol-based approaches have been implemented, with conflicting results. Extubation is defined as “the removal of the endotracheal tube” and extubation failure occurs when a patient needs a re-intubation within hours or days following a planned extubation. A failed extubation may be secondary to the incapacity to maintain oxygenation, alveolar ventilation, airway patency and protection, secretion management, or any combination of them (7). Extubation failure occurs in 3–22% of patients, independently from the underlying illness severity, with evidence that its occurrence can directly worsen patient outcomes including an increased mortality rate (8). Various clinical approaches are used to decide the best timing for extubation but no clear evidence is available over which technique is the best in children (9). The purpose of this review is to describe the most common strategies of VWe and extubation readiness tests, providing an updated view for the intensive care physicians in daily clinical practice.

## Methods

We performed a thorough literature search of Medline, Embase, Scopus, Web of Science and Cinahl to identify studies on different strategies of VWe and extubation readiness tests assessing strengths and weaknesses of each procedure. The keywords used for the research were “mechanical ventilation” AND (“extubation” OR “weaning”) AND (“infants” OR “child” OR “children” OR “pediatric”). All potentially relevant titles and abstracts were retrieved and assessed for eligibility.

## Results

### Weaning From Mechanical Ventilation

MV is divided into two phases: the maintenance phase and the weaning process. During the maintenance phase patients are usually ventilated with a synchronized modality or, if deeper sedation is required, with a controlled mode. The weaning phase usually starts when the patient is recovering from the disease which caused the intubation allowing him/her to start an inspiratory effort. The weaning process implies a transition from a full ventilatory support to a spontaneous breathing work, by gradually decreasing ventilatory parameters such as respiratory rate, positive end-expiratory pressure (PEEP) and pressure support (PS). In children, the synchronized intermittent mandatory ventilation (SIMV) with PS option is used during the weaning phase, allowing the gradual reduction of such parameters until the child is able to breath on his/her own. The weaning process is not standardized and may vary depending on different factors such as duration of MV, ventilatory muscle strength, endurance of ventilatory muscles, depth of sedation which can affect the neuromuscular conduction, and the underlying disease (16). In PICU, VWe should be a collaborative process and depends on interrelated components including clinical experience, professional judgment and autonomy, multidisciplinary team working and organizational structure (11, 13) (Table 1).

**Table 1:** Weaning from mechanical ventilation: summary of the reviewed publications.

**References**	**Country**	**Study design**	**Total sample**	**Outcome measures**	**Relevant findings**
Blackwood et al. (10)	UK	Prospective multicenter RCT	8,828	To determine if a sedation and ventilator liberation protocol intervention reduces duration of invasive MV.	Evidence of a statistically significant reduction in time to first successful extubation in the intervention group but with small size effect and uncertain clinical significance.
Duyndam et al. (11)	Netherlands	Prospective	424	To determine if the use of a nurse-driven ventilation weaning protocol in a PICU can shorten: *(i)* median duration of ventilation, *(ii)* length of PICU stay, *(iii)* compliance rate, *(iv)* rates of adverse events, *(v)* reintubation rate, *(vi)* extubation rate during nights.	Duration of ventilation does not differ between Pre-test and Post-test. Length of PICU stay is not shorter in the Post-test period. Compliance is significantly higher in the Post-test period. Adverse events were comparable. Reintubation rate is not significantly different between the Pre-test and Post-test periods. Extubation rate during nights is higher in the Post-tests period but not significantly different. So, implementation of a nurse-driven weaning protocol does not result in a significantly shorter duration of invasive MV but is safe and successful. Reintubation rate does not significantly increase compared with usual care.
Ferreira et al. (12)	Brazil	Prospective RCT	110	To evaluate usefulness of a SBT for predicting extubation success and PICU length of stay in pediatric patients in the postoperative period after cardiac surgery compare to a physician-led weaning	Population undergoing a SBT postoperatively has greater extubation success and shorter PICU length of stay compare to those weaned according to clinical judgment.
Al-Faouri et al. (13)	Jordan	Pre-test and Post-test quasi-experimental design	135	To determine the effect of educational interventions for nurses on the success of weaning trials, ventilation period and reintubation incidence for mechanically ventilated patients. Parameters evaluated: failed trials, reintubation incidence, ventilation period between two groups Pre-test and Post-test.	Failed trials are less in the experimental group. Reintubation incidence is less in the experimental group. Even though the mean score of the ventilation period is less in the experimental group, there is no significant difference between the experimental group and the control group. So educational interventions for nurses based on protocol have a significant impact on reducing incidence of failed trials and reintubation.
Ko et al. (14)	USA	Retrospective	62	To determine the ability of traditional weaning parameters to predict extubation failure in neurocritical patients (rapid shallow breathing index, minute ventilation, respiratory rate, negative inspiratory force, tidal volume and PaO2/FIO2 ratio).	None of individual weaning parameters predict extubation failure. Combination of weaning parameters do not allow prediction of extubation failure.
Jin et al. (15)	Korea	Prospective study with an historical control group	41	To evaluate, in critical ill children requiring MV, the efficacy of a sedation protocol and a “COMFORT” scale in assessing optimal sedation and how they can affect MV, length of stay in PICU, total amount and duration of sedatives and withdrawal symptoms.	Decrease in total usage of sedatives and analgesics, in duration of MV and PICU stay and in the development of withdrawal symptoms. Total duration of sedation tends to decrease. A protocol-based sedation with the “COMFORT” scale may benefit children requiring MV.
Noizet et al. (16)	France	Prospective	54	To evaluate the ability of spontaneous respiratory rate, pediatric rapid shallow breathing, occlusion pressure (ROP) and maximal inspiratory pressure during an occlusion test (Pimax) and endurance indices in predicting weaning outcome. To determine whether a combined index could enhance the ability to predict weaning success.	Best single index is ROP, best combination of indices is: (0.66 × ROP) + (0.34 × Pimax). Best endurance index is TTI2-tension time index obtained from airway pressure. Best model is: (0.6 × ROP) – (0.1 × Pimax) + (0.5 × TTI2). Combined index is of modest value in predicting weaning outcome.
Randolph et al. (17)	USA	Prospective RCT	182	To evaluate a weaning protocol instead of standard care for infants and children requiring MV support and whether a volume support weaning protocol using continuous automated adjustment of pressure support by the ventilator (i.e., volume support ventilation) is superior to manual adjustment of pressure support by clinicians (i.e., pressure support ventilation). To evaluate the performance of a set of extubation criteria on extubation success or failure and to study the relationship between sedative use during weaning and respiratory outcomes.	No significantly difference between groups for extubation failure rate and median duration of weaning among weaning successes. Male children more frequently failed extubation. Sedative use in the first 24h of weaning influences extubation failure and, among extubation successes, duration of weaning. So, weaning protocols do not significantly shorten the duration of weaning.

*MV, mechanical ventilation; PICU, pediatric intensive care unit; Pimax, maximal inspiratory pressure; ROP, rapid occlusion pressure; RCT, randomized controlled trial; SBT, spontaneous breathing trial*.

#### Monitoring During Weaning From MV

During ventilation, it is mandatory to constantly assess the correct timing for the transition between the two abovementioned phases: clinical factors such as the level of consciousness and sedation, the ability to clear the airways with effective cough and the absence of excessive secretions should guide any decision. Patients can also be considered eligible for the weaning phase when hemodynamically stable, with adequate oxygenation and gas exchange within acceptable parameters: usually FiO_2_ <50%, PaO_2_ >60 mmHg, PEEP <5–8 cm H_2_O, pH >7.25 (19, 20). After every ventilation parameter change, it is pivotal to monitor the patient's response in term of work of breathing, level of oxygenation, both clinically and with the aid of blood-gases.

#### Weaning Protocols and Techniques

While in adults, protocol-based weaning approaches are well established (20, 21), in pediatrics no protocol-based approach over a physician individualized decision has demonstrated a clear superiority. Schultz and colleagues found a reduction in the time spent in the weaning phase in the ”protocol-driven” patients compared to the ”clinician-driven” ones, even if no difference in the total duration of MV and number of complications were observed between the two groups (22). Randolph and colleagues found that weaning protocols did not significantly shorten duration of weaning in pediatric patients who had already failed one extubation (17). Nevertheless, the implementation of a sedation protocol was associated with a significant decrease in the total dose of sedatives, incidence of withdrawal symptoms, length of MV and ICU stay (15, 23). Ferreira and colleagues reduced the length of MV using a strict weaning protocol evaluating daily respiratory function coupled with echocardiographic assessment of the heart function on minimal ventilatory support (12). Minimal sedation is often feasible in adults mainly due to patient's cooperation and the early transition to tracheostomy, which is usually postponed in children. Children need to be maintained with a deeper level of sedation because of possible agitation and less tolerance to intubation. Blackwood et al. recently published a multicenter study comparing the use of a sedation and weaning protocol to the standard care including many centers (28 PICUs) and a significant number of patients (8,843 children) (10). The use of the protocol showed a significant reduction in the duration of MV to first successful extubation compared to usual care. Nonetheless, effect size was small and clinical significance uncertain considering that this approach seemed to require a huge economical and organizational investment. Despite that, the implementation of such a protocol may offer several advantages: greater involvement of nurses in ventilation, weaning and sedation assessment, a more standardized approach to ventilation and weaning and overall, a closer attention to the patient's needs.

#### Special Populations

Critically ill children affected by cardiac and neurological disorders may need special attention in the weaning process, carrying some specific features compared to the general pediatric population. Children after cardiac surgery have Pre-existing co-morbidities which may increase the severity of their conditions. Moreover, this subset of patients usually undergoes a thoracotomy/sternotomy, which alters chest wall integrity, or a cardiopulmonary bypass, that triggers a systemic inflammatory response which may result in end-organ dysfunction. Furthermore, MV itself, altering the labile equilibrium of the intrathoracic pressures may have adverse effects on hemodynamic. Also, the presence of pulmonary hypertension, a coexisting congenital syndrome or deep hypothermic circulatory arrest were all described as independent predictors of extubation failure (18).

In neurocritical care patients the success of weaning from MV depends on the underlying neurological condition: patients affected by acute syndromes/illnesses could have spontaneous recovery, while those affected by neurodegenerative diseases are predictably difficult to wean, and may require tracheostomy and possibly subsequent home ventilation. Furthermore, positive fluid balance, secondary to high amounts of intravenous fluids administered to maintain an adequate cerebral perfusion may complicate the weaning process (14).

#### Nurse-Driven Protocols

The role of nurses may be pivotal for an effective decision-making in VWe due to their active presence at the patients' bedside 24 h per day. The introduction of protocol-based weaning parameters associated with a strong training of the nurses on ventilatory management are the basis for the hypothesis that a VWe nurse-driven protocol could optimize this process. Until now, however, the application of a nurse-driven weaning protocol has not demonstrated a clear association with a reduction of weaning time and did not lead to a shorter length of MV (13). Nevertheless, the use of a nurse-driven weaning protocol is considered safe and showed a reduction of the incidence of reintubation rate compared with usual care (11). Further studies are still needed but it seems that nurse-driven protocol could upgrade the performance of PICU's nurses, improve quality of care and reduce risks for patients.

### Extubation Readiness/Failure

#### Clinical Practice and Monitoring

Readiness for extubation implies that the VWe process is almost completed; the patient should be assessed for the ability to produce effective respiratory breaths, protect the airways with intact and valid reflexes, and have a stable hemodynamic (19).

Multiple tests and indices are available, but a consensus on the best practice is still lacking (Table 2). In a work of Faustino et al., an Extubation Readiness Test (ERT) was applied to more than 1,000 children intubated for acute respiratory failure. The chances of successful extubation were greater if patients maintained the following parameters for 2 h: oxygen saturation >95%, exhaled tidal volume (VT) >5 ml/Kg and normal respiratory rate for age (27). Post extubation upper airway obstruction due to subglottic edema and stenosis could be a complication, not so rare in the pediatric population, often requiring reintubation.

**Table 2:** Extubation readiness/failure: summary of the reviewed publications.

**References**	**Country**	**Study design**	**Total sample**	**Outcome measures**	**Relevant findings**
Krasinkiewicz et al. (24)	USA	Retrospective	427	To evaluate extubation readiness practices and to identify barriers to extubation in pediatric patients who passed an extubation readiness test.	Variation in extubation readiness practices leads to a significant delay in liberation from MV. Reasons for failing an extubation readiness test are lack of spontaneous breathing, being intubated <24 hrs, breathing frequency outside the target range, not meeting tidal volume goal. Documented reasons for delaying extubation: planned procedure, neurologic status, no leak around the endotracheal tube. Median time between passing ERT and extubation is 7 hrs.
Abdel Rahman et al. (25)	Egypt	Prospective	106	To evaluate diaphragmatic and lung US indices-diaphragm thickening fraction, diaphragmatic excursion and lung US score-as new parameters predictive of weaning outcome in pediatric patients.	The analyzed indices are higher in children and infants who have a successful extubation compared with the extubation failure group.
Silva-Cruz et al. (26)	Peru	Case-Control	150	To evaluate risk factors for extubation failure in the ICU using clinical and ventilatory outcomes.	Risk factors associated to extubation failure: MV >7 days, longer time in the ICU, use of sedatives >5 days
Faustino et al. (27)	USA	Secondary analysis of a randomized trial	1,042	Accuracy of extubation readiness test in predicting extubation success in children with acute respiratory failure caused by lower respiratory tract disease.	Extubation readiness test should be considered at least daily if the OI is ≤ 6. Children passing ERT have a high probability of successful extubation.
Fernández Lafever et al. (28)	Spain	Retrospective	935	To analyze the characteristics and evolution of NIV after weaning from heart surgery in children.	NIV after weaning is associated with a rate of success of 85% and is associated with a lesser need for IMV; the most common modality is CPAP and the most common interface is “nasopharyngeal tube”.
Laham et al. (3)	USA	Prospective	319	To evaluate the ability of determining extubation readiness based on physician's judgement.	Physician's judgement has a successful rate of 91% in determining extubation readiness. First planned extubation rate success is 91%. Risk factors associated with extubation failure are the days of MV, young age, Pre-extubation steroids, Post-extubation stridor. Pre-extubation blood gas results and ventilator settings are not associated with extubation outcome. Rate of successful extubation outcome is 91% with SBT and 90% without SBT.
Baranwal et al. (29)	India	RCT	124	To evaluate occurrence of clinically significant PEAO, the time lag between extubation and occurrence of PEAO, the time to recovery from PEAO among Non-reintubated patients as measured by time to achieve mWCS ≤ 2 irreversibly.	24 hrs PD reduced the incidence of PEAO of 17% and the incidence of reintubation by 50% without statistical significance. Time to recovery from PEAO in Non-reintubated patients is shorter among 24 hrs PD patients. Intubation duration >7 days and cuffed tracheal tubes are independent risk factors for PEAO.
Fioretto et al. (30)	Brazil	Prospective	108	To compare Non-invasive positive-pressure ventilation and standard oxygen therapy Post-extubation for preventing reintubation within 48 hrs in children with respiratory failure.	No differences have been found between the groups.
Ferguson et al. (31)	USA	Retrospective	755	To evaluate the performance of an extubation readiness test based on a SBT using pressure support.	Extubation readiness test is not a significant predictor of extubation success. There is no significant relation between the number of failed ERTs and extubation failure.
Gatiboni et al. (32)	Brazil	Prospective	100	To evaluate the accuracy of several ventilatory indexes in predicting successful extubation in children considering age and specific underlying disease.	No accurate indices are predictive of extubation success; there are variations of those indices depending on age, main disease and other clinical aspects. MIP, with a cutoff ≤ 50 cmH2O, is a predictor of extubation success. Lower weight is associated with extubation failure.

*24h PD, 24-h pretreatment with dexamethasone; BNP, B-type natriuretic peptide; CPAP, continuous positive airway pressure; CROP, ratio of (dynamic compliance × maximum negative inspiratory pressure × PaO2/PAO2) to respiratory rate; ERT, extubation readiness test; FiO2, fraction of inspired oxygen; FrVe, fraction of total minute ventilation provided by the ventilator; hrs, hours; ICU, intensive care unit; IMV, invasive mechanical ventilation; min, minutes; MIP, mean maximal inspiratory pressure; MV, mechanical ventilation; mWCS, modified Westley's croup score; NIV, Non-invasive mechanical ventilation; OI, oxygenation index; P0.1, negative pressure measured 0.1 secs after occlusion of the airway; PaO2, arterial oxygen partial pressure; PAO2, alveolar oxygen partial pressure; Paw, mean airway pressure; PEAO, Post-extubation airway obstruction; Pimax, maximum negative inspiratory pressure of a spontaneous breath; PIP, peak ventilatory inspiratory pressure; RCT, randomized controlled trial; RF, respiratory frequency; RSBI, rapid shallow breathing index; SBT, spontaneous breathing trial; TV, tidal volume; US, ultrasound; VE, expired minute volume; Vt/Ti, mean inspiratory flow*.

Pre-extubation variables and weaning parameters easily obtained at patient's bedside can potentially predict the relative risk of extubation failure but evidences are still controversial. Venketaram et al. showed that an increased work of breathing resulting in higher peak inspiratory pressures, a lower tidal volume or a decreased central inspiratory drive, as shown by a VT/Ti <8 mL/kg/s, may predict a higher risk of failure (37); in other studies, though, easily measured parameters such as ventilator settings, VT, respiratory rate, Pi_max_ and arterial blood gases were not considered reliable factors for extubation readiness because of their possible correlation with other clinical aspects such as the patients' age or the underlying disease (1, 3, 32).

Newth et al. described the use of the cuff leak test, which was used to predict the occurrence of post extubation stridor, especially in patients who underwent prolonged intubation or with high amounts of secretions. During the test, a cuff deflation is applied maintaining a peak inspiratory pressure in a range between 20 and 25 cm H_2_O; usually an audible air flow from the patient is expected to be heard. Various studies showed a good specificity but low sensibility of this test, so its presence may be reassuring for the physician, but a negative test -i.e., the absence of audible leak- should not delay the extubation attempt. The serial repetition of leak tests, instead, seemed to be a good predictor for extubation readiness (4).

Ultrasound (US) is also used as a Non-invasive tool for testing patients' readiness for extubation. Rahman et al. firstly introduced diaphragm thickening fraction, diaphragmatic excursion and lung US score as possible new indices for the evaluation of diaphragmatic and pulmonary status. All parameters were significantly higher in children who underwent a successful extubation. In the future US may be used with good sensitivity and specificity as an additional predictive tool and may be considered to be integrated in an ideal protocol (25).

#### Spontaneous Breathing Trials

New techniques for assessing readiness for weaning and predicting extubation success are being developed. In adult ICU the most used technique is the SBT which consists of a period of time usually between 30 and 120 min during which the patient is examined for cardiovascular and respiratory stability while ventilated on zero to minimal respiratory support, thus permitting to test the patients' ability to tolerate clinical conditions similar to that after extubation. SBT can be performed with continuous positive airway pressure (CPAP), T-piece or PS modalities (9).

During such time, patients are strictly monitored for possible signs of poor tolerance to the trial through different methods: vital signs (e.g., increase respiratory rate and heart rate), clinical signs of increased respiratory work (e.g., apnea, decreased air entry, severe retractions, tachypnea, nasal flaring), mechanical respiratory parameters (e.g., VT, minute volume, respiratory rate, dynamic compliance and muscle strength), changes in arterial blood gases, oxygen saturation and capnography. This strategy is still not routinely used in PICU and which option is the best to predict extubation success is still under debate. Farias et al. compared SBT with the use of a T-piece vs. PS of 10 cm H_2_O before extubation in children. The study was not able to demonstrate a difference in reducing the work of breathing between SBT with T-piece and SBT with PS and both strategies had controversial aspects such as the higher cost of a T-piece system, or the air leakage of uncuffed tubes when PS was applied (1). Ferguson et al. underlined the possible negative role of PS which could mask the respiratory insufficiency due to diaphragmatic disfunction and reduce the work of breathing during the test, by overcoming the resistance of small diameter tubes (31). Chavez et al. analyzed an SBT using a T-piece/CPAP system (with an anesthesia bag) to maintain functional residual capacity during the trial. In this way continuous positive pressure prevented the development of atelectasis. Ninety-two percent of patients were successfully extubated after a positive SBT, the specificity of the SBT was 37% with a negative predictive value of 50% (35).

Another open issue is the optimal duration of SBT. In most studies the length of the test is set up to 2 h but the ideal duration is still under debate with some authors suggesting a shorter time, up to 30 min (38). Despite everything, the physician's evaluation coupled with the routine use of a suitable SBT are essential to assess the correct timing of extubation readiness. It is important to notice that, most of the abovementioned signs of failure usually develop within the initial few minutes of the SBT; the patient should be closely monitored mostly in the first phases of the trial to confirm if they are able to complete it.

#### Prognostic Factors for Extubation Failure

Several factors, related to the patient or to MV, are associated with a high risk of extubation failure. Silva-Cruz et al. enrolled 150 pediatric patients in a case-control study for the detection of risk factors associated with extubation failure. The failure group presented with a longer duration of MV, PICU length of stay and number of sedatives (26). Johnston et al. analyzed 40 infants with severe acute bronchiolitis who underwent a planned extubation; patient's weight was lower in the extubation failure group (33). Krasinkiewicz et al. reported that compromised neurological status can cause delaying in extubation. In fact, these patients either received higher doses of medication for sedation and management of pain or were unable to protect their airway for neurologic causes (24). This trend was confirmed in a similar study by Mayordomo-Colunga et al., which concluded that neurological disorders may influence extubation due to pharyngeal hypotonia and inability to properly protect airway (34).

#### Corticosteroids: Adjuncts to Extubation

Upper airway obstruction is the primary cause of extubation failure in most pediatric studies, therefore efforts should be focused on avoiding tracheal inflammation and subglottic edema before extubation. With these assumptions it is not surprising that numerous studies have focused on the use of steroids. Neonatal and pediatric studies showed mixed results. A Cochrane review concluded that the use of corticosteroids to either prevent or treat stridor after extubation has not proven effective, but “consistent trends toward benefit” were noted. Intravenous dexamethasone, administered at least once prior to extubation, was the most common steroid regimen used; in the literature, however, no evidence exists over which steroid is the most effective (29, 40). In conclusion, corticosteroids seem to be beneficial for infants and children, but definitive evidence of efficacy is lacking. Moreover, the use of multiple steroid doses and administration timing before extubation are still unclear and deserve further studies.

#### Role of Non-invasive Ventilation

Use of elective-NIV in high-risk children immediately after extubation is associated with a reduced risk of reintubation compared with rescue-NIV administered when the patient is already presenting with respiratory failure (41, 42).

In children the literature is scarce, even if the number of publications is increasing, with results comparable to adults (30, 34). Similar results were found also in children after heart surgery (28).

Furthermore, studies underline the importance of monitoring parameters such as the increase of respiratory rate (RR), blood pressure and/or oxygen demand as possible risk factors of NIV failure (34). Nevertheless, Post-extubation NIV is not yet the standard of care and still need to be clarified the need for implementation in daily clinical practice.

#### Indexes to Evaluate Extubation Readiness

In adults several integrated variables and indexes have been studied to predict extubation readiness but none of them is fully integrated in pediatric clinical practice.

Rapid shallow breathing test (RSBI) (43) is defined as the ratio between respiratory rate and tidal volume (RR/VT) when the patient breaths unassisted. This test is predictive of extubation success if <105 breaths/min/L. The test was also proposed in the pediatric population, with a modified RSBI based on patient's weight (39). However, clear-cut cut-offs are still lacking and the ones proposed showed a low sensibility and specificity (19, 44).

Maximum-inspiratory-pressure (MIP) consists in a maximal voluntary inspiratory effort against an occluded airway. Data are measured using an external manometer with a unidirectional valve or the “Negative-Inspiratory-Force” function available on most ventilators with a value to predict extubation failure of <30 cm H_2_O (45). Both these methods require full patient cooperation which is challenging to obtain in children. Finally, occlusion pressure P0.1 is the value of airway pressure 0.1 sec after initiation of an inspiratory effort against an occluded airway, reflecting patient's respiratory drive. Normal values in adults are 1–2 cm H_2_O, whereas 3–4 cm H_2_O may reflect the need for a high respiratory drive to maintain adequate alveolar ventilation. Children who fail extubation, on the contrary, were found to have a lower median P0.1, probably secondary to a reduced respiratory drive (36). Adult guidelines on weaning consider these indexes as possible ancillary tests, even if often unnecessary and redundant (20). At present, they are not sufficiently accurate in pediatrics, probably due to the wide ranges of age and weights (1, 3, 32, 46).

## Conclusions

The science of ventilator weaning and extubation readiness is still an art to be refined. No definitive data are available supporting the superiority of one approach over the others. Lack of guidelines makes the weaning from ventilation a controversial process in pediatric patients with a fundamental role still played by clinical judgment. Further studies are needed to build strong literature and standardized protocols.

## Author Contributions

EP reviewed the literature, contributed to the acquisition, interpretation of the data, and drafted the manuscript. FC, MP, DV, LZ, and FZ contributed to the acquisition and interpretation of the data, drafted the manuscript, and reviewed the literature. AP critically revised the manuscript. MD drafted and critically revised the manuscript. CM conceived the idea for this review, drafted, and critically reviewed the manuscript. All the authors finally approved the manuscript. All authors contributed to the article and approved the submitted version.

## Conflict of Interest

The authors declare that the research was conducted in the absence of any commercial or financial relationships that could be construed as a potential conflict of interest.

## Publisher's Note

All claims expressed in this article are solely those of the authors and do not necessarily represent those of their affiliated organizations, or those of the publisher, the editors and the reviewers. Any product that may be evaluated in this article, or claim that may be made by its manufacturer, is not guaranteed or endorsed by the publisher.

## Abbreviations

CPAP: continuous positive airway pressure
ERT: extubation readiness test
MIP: maximum-inspiratory-pressure
MV: mechanical ventilation
NIV: Non-invasive ventilation
PEEP: positive end-expiratory pressure
PICU: pediatric intensive care unit
PS: pressure support
RR: respiratory rate
RSBI: rapid shallow breathing test
SBT: spontaneous breathing trials
SIMV: synchronized intermittent mandatory ventilation
VT: tidal volume
VWe: ventilation weaning.
