# Corrigendum on: Ventilation weaning and extubation readiness in children in pediatric intensive care unit: A review

**DOI:** 10.3389/fped.2022.1044681

**Published:** 2022-10-14

**Authors:** Elisa Poletto, Francesca Cavagnero, Marco Pettenazzo, Davide Visentin, Laura Zanatta, Fabrizio Zoppelletto, Andrea Pettenazzo, Marco Daverio, Claudia Maria Bonardi

**Affiliations:** ^1^ Department of Woman’s and Child’s Health, University Hospital of Padua, Padua, Italy; ^2^ Pediatric Intensive Care Unit, Department of Woman’s and Child’s Health, University Hospital of Padua, Padua, Italy

**Keywords:** weaning, mechanical ventilation, extubation, pediatric, children, pediatric intensive care unit

A corrigendum on Ventilation weaning and extubation readiness in children in pediatric intensive care unit: A review By Poletto E, Cavagnero F, Pettenazzo M, Visentin D, Zanatta L, Zoppelletto F, Pettenazzo A, Daverio M and Bonardi CM. (2022) Front. Pediatr. 10: 867739. doi: 10.3389/fped.2022.867739

In the published article, the first and last names were switched. The author list has been changed from Poletto Elisa, Cavagnero Francesca, Pettenazzo Marco, Visentin Davide, Zanatta Laura, Zoppelletto Fabrizio, Pettenazzo Andrea, Daverio Marco and Bonardi Claudia Maria to Elisa Poletto, Francesca Cavagnero, Marco Pettenazzo, Davide Visentin, Laura Zanatta, Fabrizio Zoppelletto, Andrea Pettenazzo, Marco Daverio and Claudia Maria Bonardi.

The authors apologize for this error and state that this does not change the scientific conclusions of the article in any way. The original article has been updated.

